# Chromosomal instability associated with adverse outcome: a case report of patient with Nijmegen breakage syndrome and rapidly developed T-NHL with complex karyotype

**DOI:** 10.1186/s13039-020-00505-2

**Published:** 2020-08-20

**Authors:** Monika Włodarczyk, Monika Lejman

**Affiliations:** 1 Laboratory of Genetic Diagnostics, Medical University of Lublin, Lublin, Poland; 2 Department of Paediatric Haematology, Oncology and Transplantology, Medical University of Lublin, Lublin, Poland

**Keywords:** Nijmegen breakage syndrome, Non-Hodgkin lymphoma, Gene, FISH, Microarray

## Abstract

**Background:**

Nijmegen breakage syndrome (NBS) is a rare genetic disorder inherited in an autosomal recessive pattern associated with an increased risk of developing lymphoproliferative disorders, mainly non-Hodgkin lymphoma (NHL) and acute lymphoblastic leukemia (ALL). NBS patients are 50 times more likely to develop malignancy than healthy controls. Moreover, in NBS, mortality rate from cancers, mainly lymphomas, is the highest among all diseases associated with excessive fragility of chromosomes.

**Case presentation:**

This work presents a patient previously diagnosed with Nijmegen breakage syndrome who rapidly developed T-NHL despite of constant medical supervision. Cytogenetic karyotype and microarray tests revealed complex aberrations, indicating enhanced chromosomal instability. Despite initial steroid therapy, the patient passed away due to multiorgan failure.

**Conclusions:**

The lack of well-established diagnostic procedures in NBS patients make it difficult to determine any therapeutic target or predictive marker. Moreover, anticancer treatment is the biggest challenge in NBS patients due to therapy-related toxicity and immunodeficiency. Our case indicates the importance of identifying parameters useful in prognosis of disease outcome, as main risk factor affecting overall survival in NBS patients is an extremely high incidence of malignancy development.

## Introduction

Nijmegen breakage syndrome (NBS) is a rare genetic disorder inherited in an autosomal recessive pattern associated with an increased risk of developing lymphoproliferative disorders, mainly non-Hodgkin lymphoma (NHL) and acute lymphoblastic leukemia (ALL) [1, 2]. Moreover, NBS patients are 50 times more likely to develop malignancy than healthy controls [3]. The disease is caused by mutations in *NBS1* gene located on chromosome 8q21. The most commonly observed NBS mutation, affecting approximately 90% of all NBS cases, is 657_661del5 in exon 6 of *NBN* gene [1, 4]. Furthermore, most of NBS patients are of Slavic origin, thus this particular alteration is called Slavic mutation [4]. NBS seems to occur worldwide, but the majority of cases were reported among Central European and Eastern European populations (Poland, Czech Republic, Ukraine) with relatively high (1/177) carrier frequency [1, 5–7].

Characteristic cellular features of the NBS include increased telomere loss and sensitivity to ionizing radiation, and chromosomal instability resulting from spontaneous chromosome aberrations, usually affecting locus on chromosomes 7 (T-cell receptor (TCR) gene cluster) and 14 (immunoglobulin heavy chain gene cluster) [4]. Among the clinical features of NBS are progressive microcephaly, dysmorphic facial features (including sloping forehead, prominent nose, small mandible, long philtrum), dysmorphic ears, mild growth retardation and immunodeficiency [5, 7]. In NBS, mortality rate from cancers, mainly lymphomas, is the highest among all diseases associated with excessive fragility of chromosomes [8, 9].

## Case report

A 4-year-old boy was admitted to Department of Genetic Diagnostics due to microcephaly, mild growth retardation and dysmorphic facial features, such as sloping forehead, large ears and prominent nose. There were no comorbidities, as well as no significant findings in the patient’s family history. To assess the somatic karyotype of patient culture of blood samples was performed under standard conditions of 37 °C and 5% CO_2_ in PB MAX Karyotyping Medium (Thermo Fisher Scientific, Waltham, MA, USA). To stop cell division at mitosis, a mitotic inhibitor (Colchicyne Solution 10 μg/μl in NBSS, Thermo Fischer Scientific, Waltham, MA, USA) was added to the cell culture. Then, Carnoy’s solution (3:1 methanol:acetic acid) was used to fixation of cells. GTG band staining was performed and the karyotype of patient was assessed using Axio Imager.Z2 microscope (Zeiss, Oberkochen, Germany) and Applied Spectral Imaging (Carlsbad, CA, USA) software. The karyotype was described according to The International System for Human Cytogenetic Nomenclature (ISCN). Cytogenetic analysis revealed normal karyotype, including no cytogenetic abnormalities involving chromosomes 7 and 14. No chromosomal instability was found in any of the chromosomes, thus further analysis was performed using molecular techniques (Sanger method, ABI 3130, Applied Biosystem, MA, USA). The patient was diagnosed with Nijmegen breakage syndrome as genetic test confirmed homozygotic deletion c.657_661delACAAA in the *NBN* gene.

After 2 years, the boy was admitted to the Department of Pediatric Hematology, Oncology and Transplantology, Medical University of Lublin, Poland, due to pneumonia. The boy reported pain lasting 3 weeks in the lower extremities and swollen submandibular nodes from a week. The patient’s condition was defined as severe, as his examination revealed leukocytosis (white blood cells = 50,000/μl), lymphadenopathy and the presence of a tumor in the mediastinum. Myelogram presented 54% blasts with T-NHL phenotype: TCRα/β– TCRγ/δ+, CD45+, CD7+, cytCD3+, CD3+, CD19dim+, CD5dim+, CD2+, CD45RA+, CD45RO+, CD8+, CD33+, CD13+, CD117dim+, CD123dim+, CD16dim+, CD11c+. Ultrasound examination of the neck revealed lymph node conglomerates on both sides with reduced echogenicity and rounded shape. Lymph node biopsy was performed for histopathological examination, which showed hyperplasia of lymphoblastic morphology. Lymphoblastic cells revealed a positive expression of CD3c, CD7, CD2, CD5, CD4, CD8, moderate CD1a, TdT, CD56 and CALLA expression, and low CD79a expression. The result indicates cortical type of T cell lymphoblastic proliferation. The boy was diagnosed with stage IV T-NHL.

**Fig. 1:**
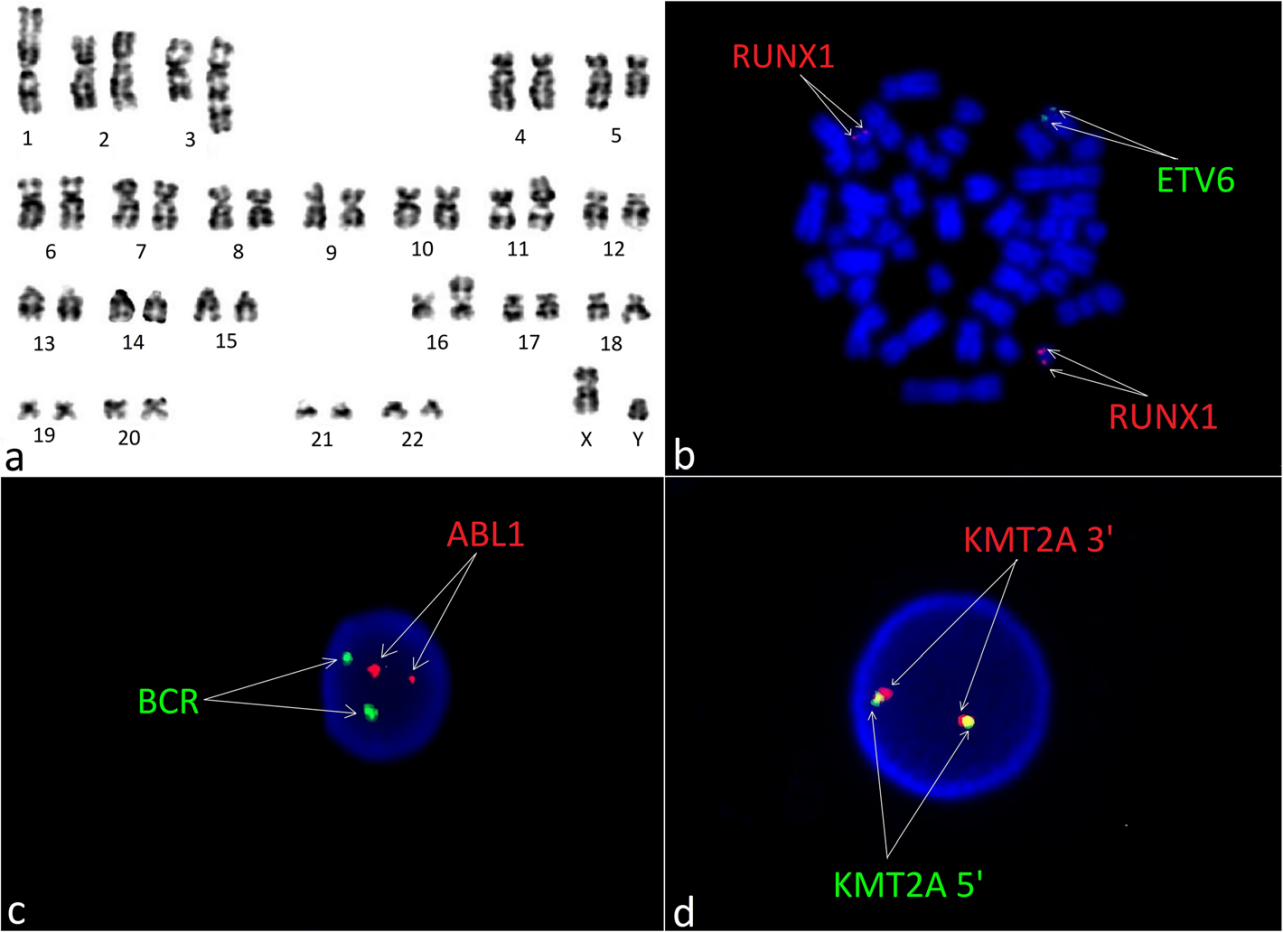
Cytogenetic analysis of bone marrow cells at diagnosis of T-NHL in 6-year-old male. (**a**) The karyogram (GTG-banding) showing complex karyotype of the patient: 45,XY,-1, dup(1)(p32p34),der(3)t(1;3)(q12;q22),der(5)t(5;10)(q21;p11),der(9)t(4;9)(q32;p21),der(11)t(1;11)(p32;p13),del(12)(p13),der(16)t(6;16)(q22;p13)[10]/46,XY[15] (**b,c,d**) Results of FISH tests with probes: ETV6/RUNX1, BCR/ABL1 and KMT2A. FISH was performed on metaphases and interphase nuclei using probes (Cytocell Ltd., Oxford Gene Technology, Cambridge, United Kingdom) according to the manufacturer’s recommendations. Images were captured by an Olympus BX41TF microscope equipped with a Jenoptik camera and analysed with Isis Software (MetaSystems)

**Fig. 2:**
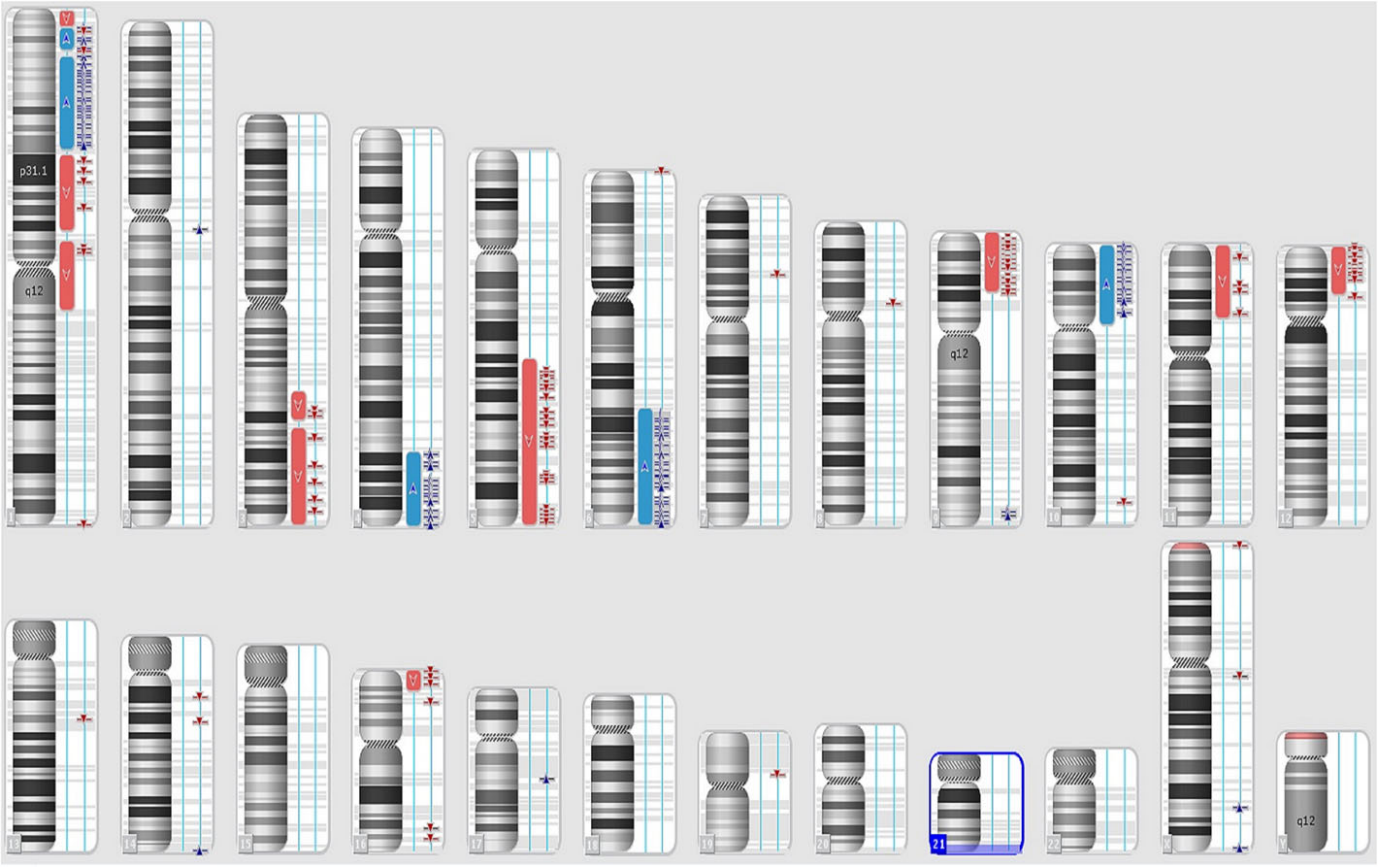
Karyoview from microarray test and a scheme presenting chromosomal aberrations in patient. Microarray results revealing partial gains of overlapping regions on chromosomes 1p 8,931,529-67,365,806 bp (1p36.23-p31.3), 4q 155,500,158-190,957,473 bp (4q31.3-q35.2), 6q 115,144,178-170,919,482 bp (6q22.1-q27) and 10p 100,026-38,258,848 bp (10p15.3-p11.1). Moreover, regions of overlap of deletions were also found on chromosomes 1p 849,466-8,096,240 bp (1p36.33-p36.23) and 1 70,493,564-145,289,186 bp (1p31.12-q21.1), 5q 100,821,228-180,719,789 bp (5q21.1-q35.3), 9p 203,861–28,849,504 bp (9p24.3-p21.1), 11p 230,615-35,363,338 bp (11p15.5-p13), 12p 173,786-22,885,159 bp (12p13.33-p12.1) and 16p 85,880-10,023,421 bp (16p13.3-p13.2). Asterisks correspond to deletion (red colour), duplication (blue colour) and loss of heterozygosity (purple colour)

**Fig. 3:**
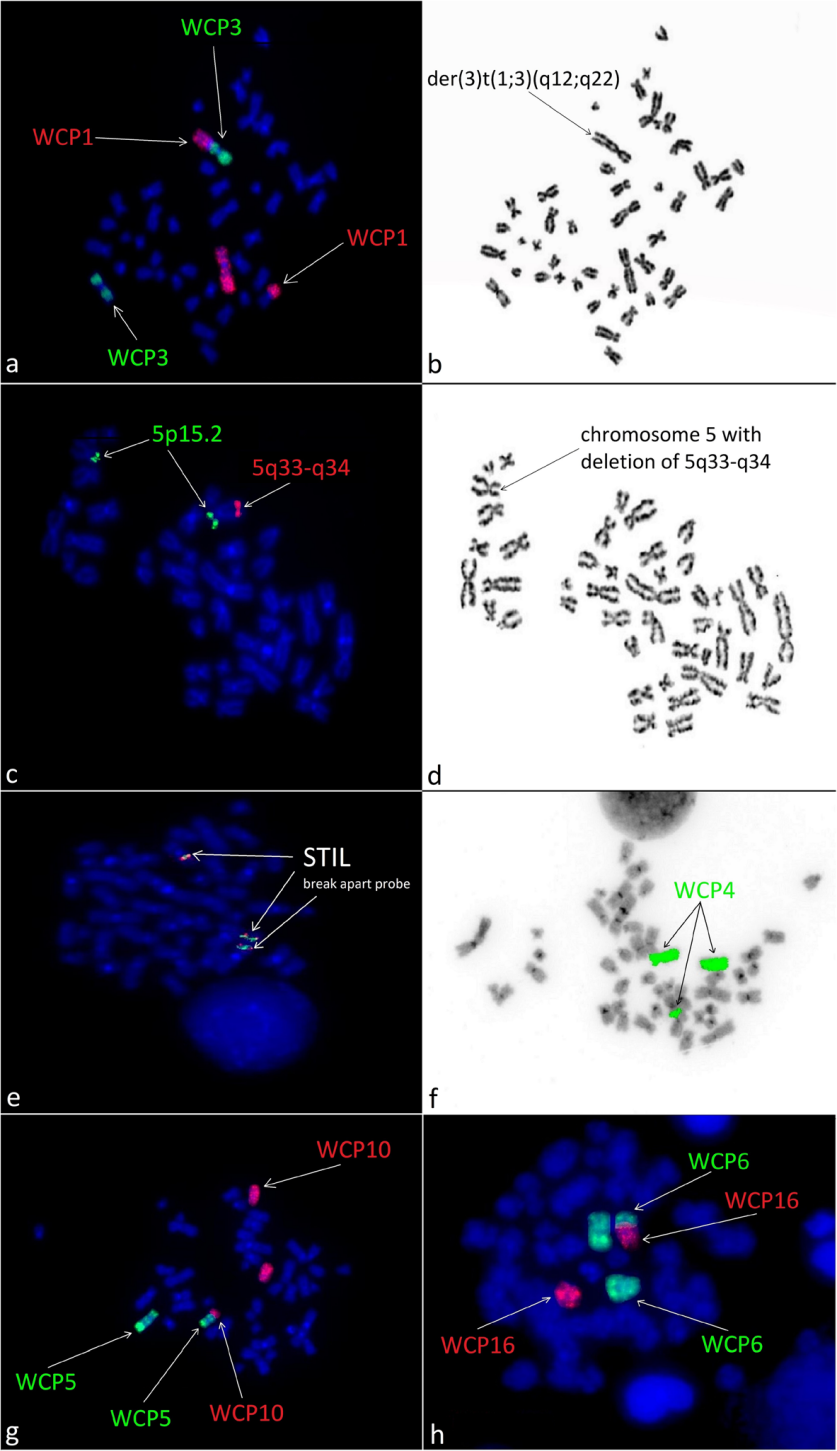
Images of the FISH results revealing chromosomal aberrations. (**a**) Image of the FISH results with the whole chromosome painting (WCP) 1 and 3 probes (Cytocell Ltd., Oxford Gene Technology, Cambridge, United Kingdom) revealing t(1;3). (**b**) Chromosome analysis demonstrating derivative chromosomes 3 der(3)t(1;3)(q12;q22) and chromosome 1. (**c**) Image of the FISH results with the LSI CSF1R/D5S23, D5S721 Dual Color probe (Vysis, Abbot Molecular, Illinois, USA) revealing del(5q33-q34). (**d**) Chromosome analysis demonstrating abnormal chromosome 5 with deletion of 5q33-q34. (**e**) Image of the FISH results with the STIL Break Apart Probe (Empire Genomics, New York, USA) revealing *STIL* duplication. (**f**) Image of the FISH results with WCP4 probe (Cytocell Ltd., Oxford Gene Technology, Cambridge, United Kingdom) revealing t(4;9). (**g**) Image of the FISH results with WCP5 and WCP10 probes (Cytocell Ltd., Oxford Gene Technology, Cambridge, United Kingdom) revealing t(5;10). (**h**) Image of the FISH results with WCP6 and WCP16 probes (Cytocell Ltd., Oxford Gene Technology, Cambridge, United Kingdom) revealing t(6;16). Images were captured by an Olympus BX41TF microscope equipped with a Jenoptik camera and analysed with Isis Software (MetaSystems)

**Fig. 4:**
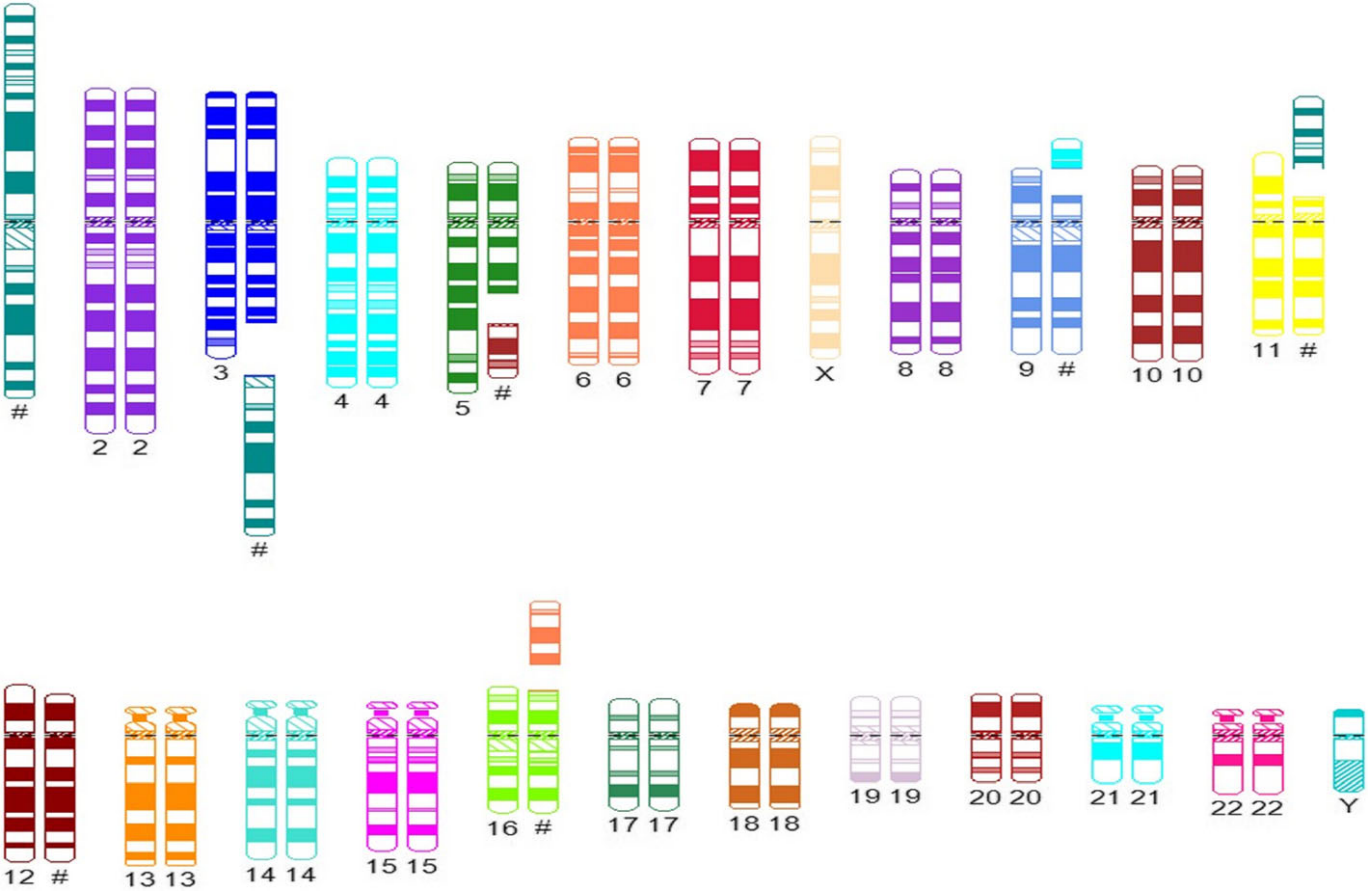
The scheme of chromosomal aberrations in patient based on cytogenetic analysis, microarray tests and FISH results prepared using CyDAS software (http://www.cydas.org/OnlineAnalysis/, Duesseldorff, Germany). Hash represents derivative chromosomes

Moreover, 24-h unstimulated cell culture of bone marrow samples in standard conditions in MAX Bone Marrow Medium (Gibco, Thermo Fischer Scientific, Waltham, MA, USA) was performed to assess the somatic karyotype of patient. GTG band staining (Fig. 1a) and fluorescence in situ hybridization (FISH) test were performed with the use of probes: BCR/ABL1, KMT2A, ETV6/RUNX1 (Vysis, Abbot Molecular, Illinois, USA). The arrangement from ETV6/RUNX1 probe suggested *ETV6* deletion (Fig. 1b). The arrangement of signals from other probes used was correct (Fig. 1c and d). Cytogenetic karyotype revealed many aberrations, but it was difficult to recognize and assess correct result from karyotype. Thus, microarray analysis was performed to improve genetic diagnosis (CytoScan HD, Applied Biosystems, part of Thermo Fischer Scientific, Waltham, MA, USA). Tests revealed additional alterations in the form of gained copies (4q32-q35, 6q22-q27, 10p11-p15) and loss regions (9p21-p24, 5q21-q35) (Fig. 2). Cytogenetic and microarray results were partially confirmed by FISH tests (Fig. 3). Finally cytogenetic result was the following: 45,XY,-1,dup(1)(p32p34),der(3)t(1;3)(q12;q22),der(5)t(5;10)(q21;p11),der(9)t(4;9)(q32;p21),der(11)t(1;11)(p32;p13),del(12)(p13),der(16)t(6;16)(q22;p13)[10]/46,XY[15] (Fig. 4 and Table 1). Despite initial steroid therapy, the patient passed away after 21 days due to multiorgan failure. Medical history of patient revealed that he was not exposed to radiation or any genotoxic agents since NBS diagnosis.

**Table 1:** Cytogenetic and molecular features of chromosomal instability in described patient

Chromosome aberration	Cytogenetic test	Cytobands	Microarray nomenclature	Cytoregions (OMIM genes)	FISH confirmation	Probe
1		1p36.33p36.23	arr[hg19] 1p36.33p36.23(849,466-8,096,240)x1–2	*CAMTA1, ERRFI1, MIB2, RPL22, PRDM16, DVL1*		
	dup(1)?(p32p34)	1p36.23p36.13	arr[hg19] 1p36.23p36.13(8,931,529-19,215,840)x2–3	*PIK3CD, PRDM2, SDHB, CASP9, MTOR, MTHFR, ENO1*	nuc ish (STILx3)	LSI STIL Dual Color, Break Apart Rearrangement Probe (Empire Genomic)
		1p36.12p31.3	arr[hg19] 1p36.12p31.3(23,146,680-67,365,806)x2–3	*MUTYH, RPS6KA1, STIL, IL22RA1, LCK, PTCH2, PPP1R8, JAK1, CSF3R, JUN, SFPQ, CITED4, CDKN2C, RPA2, MPL, YBX1, CLIC4, TSPAN1, TAL1, COL16A1, HNRNPR, MDS2, RSPO1, PRDX1, EPS15, CDC20, PDZK1IP1*		
		1p31.1p21.1	arr[hg19] 1p31.1p21.1(70,493,564-106,636,210)x1–2	*BCL10, SEP15, GLMN, RPL5, TGFBR3, GBP1, LPHN2, GFI1*		
		1p13.2q21.1	arr[hg19] 1p13.2q21.1(111,894,976-145,289,186)x1–2	*NOTCH2, RHOC, SLC16A1, HIPK1, FAM46C, WDR77, NRAS, BCL2L15, REG4, RAP1A, VTCN1, PDE4DIP*		
3	der(3)t(1;3)(q12;q22)	3q22.1q24	arr[hg19] 3q22.1q24(133,476,890-146,949,828)x1–2	*ATR, FAIM, RNF7*	ish der(3)t(1;3)(wcp3+,wcp1+)	WCP1 WCP3 (Cytocell)
		3q25.1q29	arr[hg19] 3q25.1q29(151,583,903-197,851,986)x1–2	*PLD1, BCL6, MME, PIK3CA, MUC4, TNFSF10, EIF4A2, DLG1, RAP2B, MECOM, TBL1XR1, LPP, GMPS, PAK2, MECOM, MLF1, RARRES1, SOX2, TFRC*		
4	invisible	4q31.3q35.2	arr[hg19] 4q31.3q35.2(155,500,158-190,957,473)x2–3	*ING2, NPY1R, FAT1, SORBS2*		
5	der(5)t(5;10)(q21;p11)				ish der(5)t(5;10)(wcp5+,wcp10+)	WCP5 WCP10 (Cytocell)
		5q21.1q35.3	arr[hg19] 5q21.1q35.3(100,821,228-180,719,789)x1–2	*RANBP17, SNX2, ACSL6, TGFBI, ITK, PTTG1, TSLP, LOX, ARHGAP26, SPINK7, IRF1, NR3C1, APC, TNIP1, NSD1, CSNK1A1, NPM1, GNB2L1, NKX2–5, AFF4, MAPK9, FNIP1, EBF1, CSF1R, TLX3, IL3, HDAC3, EGR1, PDGFRB*	del(5)(q33)(D5S23,D5S721+,CSF1R-)	LSI 5q33q34 (CSF1R)Orange/D5S23,D5S721Green Probe Set (Vysis)
6	invisible	6q22.1q27	arr[hg19] 6q22.1q27(115,144,178-170,919,482)x2–3	*ECT2L, PLAGL1, hsa-mir-548a-2, MLLT4, MYB, IGF2R, AHI1, TNFAIP3, BCLAF1, FGFR1OP, RNF217-AS1, CTGF, CEP85L, AKAP12, CITED2, RNASET2, THBS2, LATS1*		
9	der(9)t(4;9)(q32;p21),	9p24.3p21.1	arr[hg19] 9p24.3p21.1(203,861–28,849,504)x1–2	*CDKN2A, IFNA1, RLN2, MLLT3 (AF9), SH3GL2, CDKN2A, TEK, JAK2, MTAP, KDM4C, PTPRD, PSIP1, RFX3, CDKN2B, MLLT3*		WCP 4 (Cytocell)
10	invisible	10p15.3p11.1	arr[hg19] 10p15.3p11.1(100,026-38,258,848)x2–3	*BMI1, MLLT10, ABI1, KLF6, GATA3, NET1, AKR1C3, MRC1*		
11	der(11)t(1;11)(p?32;p13)	11p15.5p13	arr[hg19] 11p15.5p13(230,615-35,363,338)x1–2	*HRAS, PAX6, KIAA1549L (C11orf41), WT1, MUC2, CD44, CD151, EIF3F, LMO1, CARS, HTATIP2, FANCF, RRM1, LMO2, MUC6, NUP98*	der(11)t(1;11)(wcp1+,wcp11+),	
12	del(12)(p13)	12p13.33p12.1	arr[hg19] 12p13.33p12.1(173,786-22,885,159)x1–2	*FOXM1, ERC1, KDM5A, ING4, ATF7IP, EPS8, ETV6, MIR200C, RECQL, BCL2L14, CCND2, GUCY2C, ZNF384, CDKN1B, KLRK1, VWF, CD9, ETNK1, GABARAPL1*	nuc ish(ETV6x1,RUNXx2)	LSI ETV6(TEL)/RUNX1(AML1) ES Dual Color Translocation Probe Set (Vysis)
16	der(16)t(6;16)(q22;p13)	16p13.3p13.2	arr[hg19] 16p13.3p13.2(85,880-10,023,421)x1–2	*TRAP1, AXIN1, CREBBP, PKD1, TSC2, USP7*	der(16)t(6;16)(wcp6+,wcp16+)	WCP6 WCP16 (Cytocell)

## Discussion and conclusions

*NBN* gene encodes for a protein (nibrin), which is a part of the Mre11/Rad50/NBN (MRN) nuclear protein complex. MNR function is crucial for DNA repair (especially double strand breaks, DSBs), recombination processes and checkpoint arrest [10, 11]. Maintaining genome integrity is important for any organism, as the resulting modifications are associated with an increased risk of mutagenesis or carcinogenesis. In physiological conditions, double strand breaks are observed during DNA replication and meiotic recombination and in the processes of development of acquired immunity, as DNA DSBs occur in V(D) J recombination during early B and T cells differentiation and immunoglobulin class switch in mature B cells [4, 7].

*NBN* mutation results in the fragmentation of nibrin into two nonfunctional parts: the 26 kDa N-terminal fragment and the 70 kDa fragment, which retains the residual nibrin function [4]. Homozygous carrier of this mutation is associated with very early incidence of lymphomas, sarcomas and gliomas [4, 12, 13]. However, in Slavic populations, heterozygous carriers of the 657del5 mutation or the molecular variant R215W of the *NBN* gene are often observed [1]. Population studies revealed that heterozygous carriers of the *NBN* mutation are also at increased risk of developing lymphoproliferative cancers [1, 14].

Early diagnosis of NBS is crucial as it prevents from severe recurrent infections and unnecessary exposure to radiation during diagnostics procedures [4, 7]. Due to the evolution of monoclonal gammopathy towards lymphoproliferative disorders in immunocompromised patients, monitoring of this parameter may be useful in determining the risk of developing malignancies in NBS patients [4]. Nevertheless, an improvement of immune system is needed to avoid further malignancies in patients with NBS and NHL.

From the moment of diagnosis, the patient was under constant medical supervision, and yet he developed advanced NHL as the consequence of extremely high chromosomal instability. Predisposition to malignancies, including lymphoid malignancies, is associated with chromosomal instability, as NBS patients have 250-fold risk of developing lymphomas [1, 4]. Several non-specific symptoms, such as nodal enlargement and fever are thought to be connected with infection disease in NBS patients. Therefore, in NBS cases, advanced stages of lymphomas with multiorgan involvement are commonly observed [14, 15]. High incidence of lymphoma relapse, reduced treatment tolerance and delayed diagnosis of lymphoproliferative disorders in NBS patients are the cause of poor prognosis [15, 16]. The distribution of B and T cell lymphoma in NBS patients was described in several studies to date [17]. We present for the first time a case of patient with NBS who developed T-NHL in relatively short time despite medical geneticists’ supervision.

Chromosomal instability is associated with development of complex genetic markers in pre-cancer cells. Moreover, simultaneous acquisition of structural chromosomal aberrations and mutation enables tumor evolution, thus leading to poor outcome [18]. Despite the karyotype of NBS patients is generally normal, a lot of abnormalities in the form of aneuploidies, structural rearrangements and marker chromosomes may be observed in 10–60% of cells [4].

As *NBN* mutations affects maturation and function of T and B cells, NBS patients are high susceptible to infections, mostly involving respiratory system [4]. Moreover, due to bone marrow failure, severe infections, cardio- and nephrotoxicity, some forms of chemotherapy (including anthracyclines methotrexate and alkylating agents) and radiotherapy should be limited in the treatment of patients with NBS [4, 19]. Hematopoietic stem cell transplantation seems to be a last treatment option in NBS patients in whom standard chemotherapy protocols have failed [19].

The lack of well-established diagnostic procedure in NBS patients make it difficult to determine any therapeutic target or predictive marker [19]. Furthermore, anticancer treatment is the biggest challenge in NBS patients due to therapy-related toxicity and immunodeficiency.

The main risk factor affecting overall survival in NBS patients is an extremely high incidence of malignancy development. Most of NBS patients die in first decade of life due to unsuccessful cancer treatment, thus novel therapeutic intervention development is of great clinical importance [4, 19]. Therefore, our case indicates the necessity of identifying parameters useful in the prognosis of NBS patients.

## Data Availability

The datasets generated and/or analysed during the current study are available in the Gene Expression Omnibus (GEO) repository, https://www.ncbi.nlm.nih.gov/geo/query/acc.cgi?acc=GSE148229.

## Abbreviations

NBS: Nijmegen breakage syndrome
NHL: Non-Hodgkin lymphoma
ALL: Acute lymphoblastic leukemia
TCR: T-cell receptor
FISH: Fluorescence in situ hybridization
DSB: Double strand breaks
